# Supporting Acquisition of Spelling Skills in Different Orthographies Using an Empirically Validated Digital Learning Environment

**DOI:** 10.3389/fpsyg.2021.566220

**Published:** 2021-04-06

**Authors:** Heikki Juhani Lyytinen, Margaret Semrud-Clikeman, Hong Li, Kenneth Pugh, Ulla Richardson

**Affiliations:** ^1^University of Jyväskylä, Jyväskylä, Finland; ^2^University of Minnesota, Minneapolis, MN, United States; ^3^Beijing Normal University, Beijing, China; ^4^Haskins Laboratories, Yale University, New Haven, CT, United States

**Keywords:** reading acquisition, dyslexia, intervention, digital learning game, GraphoLearn technology, GraphoGame, writing system, orthography

## Abstract

This paper discusses how the association learning principle works for supporting acquisition of basic spelling and reading skills using digital game-based learning environment with the Finland-based GraphoLearn (GL) technology. This program has been designed and validated to work with early readers of different alphabetic writing systems using repetition and reinforcing connections between spoken and written units. Initially GL was developed and found effective in training children at risk of reading disorders in Finland. Today GL training has been shown to support learning decoding skills among children independent of whether they face difficulties resulting from educational, social, or biological reasons.

## Introduction

Our experience with teaching alphabetic reading and spelling in a transparent writing system (Finnish) provides a basic model of how digital training can be used when instructing less transparent writing systems such as English. Our work demonstrates how learning can occur almost as rapidly in English as in Finnish.

The GraphoLearn technology (GL) is a digital learning environment that assists with literacy acquisition that can be applied globally. GL grew out of the Jyväskylä Longitudinal Study of Dyslexia (JLD), which followed children at familial risk for dyslexia from birth to adulthood (Lyytinen et al., 2006, 2009). The main goal of the JLD means to identify them prior to school entrance and learn to understand how to help children at risk for dyslexia [Lyytinen et al., 2007; Lyytinen, 2010; for a most recent summary, see Lyytinen et al. (2015b)]. Subsequently, the work developed GraphoGame (GG) to support at-risk children outside Finland with the research version called GraphoLearn (GL) adopted for several additional languages beyond Finnish. Once empirical effectiveness has established a specific language, the game is made available to the general public as GraphoGame (GG). Empirical validation for GL has been established in more than 20 languages (for publications on which this all is based, see https://info.grapholearn.com/research/publications/).

Upon completion of GL training, learners remember the connections between units of spoken and written language. In orthographically consistent written languages, students learn the sound represented by each letter (or graphemes of several letters). In contrast, for languages with less consistent orthographies at the phoneme–grapheme level (i.e., English), letters do not consistently represent the same sound requiring additional instruction. Association learning requires the use of consistent connections to be effective; thus, for the connections between oral and written English to be appropriately stored, substantially larger units utilizing rimes are required [see Ziegler and Goswami (2005)]. Using this approach, the GraphoLearn method has been empirically validated to support the development of reading and spelling skills in English (e.g., Kyle et al., 2013; Ahmed et al., 2020). Based on this research, we hypothesized that there are universal rules that are appropriate to learning, irrespective of the specifics of the writing system.

Richardson and Lyytinen (2014) describe the basic principles of how the GraphoLearn (GL) technology can be used. The successful effects of training basic reading and spelling skills have been documented in several research studies conducted in different countries and alphabetic orthographies varying in transparency. In countries with effective school instruction, GL is meant to help early learners who have reading/spelling acquisition originating from biological etiologies (dyslexia) as well as other compromising conditions (e.g., poor or no instruction, poverty, and lack of social support). This essential connection-building operation has been empirically validated to apply to almost any language. Longer instructional times are required for children with severe learning difficulties using GL. Moreover, typically developing children can acquire fluent reading skills in less time than usual instructional techniques.

Recent GL studies reveal that severe dyslexia is very difficult to overcome when intervention is started after the child has been affected by the related failures during the first semester (Ronimus et al., 2019). Thus, the optimal starting time is when children enter school. When adults motivate the child to use GraphoGame/GraphoLearn, successful learning is more likely to occur (McTigue et al., 2019). We have observed that the very same GL versions implemented with appropriate language content help learners in Africa [for a most recent review of studies, see Lyytinen et al. (2019a,b)] where instruction is not always optimal and who in Latin America are failing to learn to read due to insufficient social support (Ecochard, 2015), as the game keeps them engaged in training long enough to reach the goal.

## Acquisition of Basic Reading Skills in Transparent Writing Environments

As noted, Finnish orthography has a straightforward relationship to spoken Finnish where a particular sound is consistent in all contexts. Thus, Finnish is extremely easy to master once the sounds for the 23 Finnish letters are learned. In Finland, approximately half of children learn to read before entering school without formal instruction. For the remaining children who do not readily learn to read, the use of the beginner's version of the GraphoGame (called Ekapeli Alku in Finland) helps them stay in pace with their classmates. Even a brief exposure to the program often results in significant reading gains (Richardson et al., 2011). The optimal use of this program includes starting at the time the learner enters school, when children tend to be most interested in developing their reading skills, which has been documented using stored data (i.e., detailed learning logs) from all users.

The main idea of the game is to motivate the learner to choose from (2–8) alternative letters (or letter sequences) the one which represents the sound the player is hearing at the same time through headphones. The trials these children play are analyzed using the correct/incorrect selections of the written item for each target sound (phoneme, syllable, or word). Additionally, the response times the learner needs for selecting the written item are stored to find out how the automatization is developing.

Using the collected logs, it has been possible to see that during a single day one third of the age cohort in Finland has been using the program. This number of users is observed close to the time children enter primary school. The use of the game also supports fluency of reading and spelling (Heikkilä et al., 2013), which makes it understandable that some of the players may have been second graders. Practically all Finnish-speaking children are accurate (but not necessarily fluent) readers already during the first school semester. However, even during the later months of the 1st grade as well as during the 2nd grade some children are still interested in using the program for acquiring more fluency.

While relatively short use of the game helps most children, those who have severe difficulties due to biological reasons need more time to overcome their problems. Most children using the GL in transparent writing environments in the developed world get sufficient help, preventively using it to follow the mainstream learning curve in the basic reading/spelling skills. This finding is true concerning children with dyslexia if GL is used when the child first begins school and the game is used for 10–15-min sessions (preferably more than one) per day for several weeks. Children facing severe difficulties most often require continuation until the third grade especially if the game is used only relatively infrequently (i.e., a few short sessions per week). This program is most effective when the game is used in the context of face-to-face remedial teaching. We have found that remedial teaching is strongly enhanced when the game is utilized as an addition to that remediation. Our studies have revealed that if the training game is used for at least a quarter of the time (10–15 min) the child is in the special education session, learners improve their spelling skills to the level of the mainstream children by the end of the third grade (Saine et al., 2010, 2011, 2013).

Our studies provide strong evidence that support with GL needs to start early in the primary school grades in order to be optimally effective. If learners start the program too early, the risk increases that the child may not be motivated to continue using it; they become bored with the task as too many repetitions are required before the goal is reached. This could be because many children with dyslexia have delayed brain development reflected by accompanying delayed development of spoken language (e.g., Lyytinen et al., 2015a); thus, starting this game too early may not be effective because the child is not ready yet to learn the connections without very numerous repetitions.

McTigue et al. (2019) conducted a metastudy of GraphoGame which provides additional guidance for teachers on the use of the game. These suggestions work in whatever orthography children are getting help *via* GraphoLearn technology, particularly with children with dyslexia.

Well-timed, the combination of GG and remedial teaching is highly effective with children with dyslexia because the game is highly enjoyable and focuses specifically on the individuals' “bottlenecks,” areas which still need training (at an individual level), without spending precious time on irrelevant activities (i.e., which are not directly supporting the connection building). Decoding bottlenecks are overcome by continuing the repetition of tasks by concentrating on each aspect of difficulty separately (e.g., using so-called minimal-pair training when needed). Only 20% of the trials are chosen to be focused on contents where the learner tends to make repeatedly incorrect choices (i.e., getting thus negative feedback), to keep playing enjoyable. The crux of the program rests on positive feedback, to motivate the learner to continue practicing the task until the goal is reached. GL's effectiveness results from the real-time adaptation to every individual's actual needs rather than on traditional remedial activities that do not directly build the still compromised aspects of literacy-related skills for children who require remediation.

Thus, it is easy to understand that spelling has been found to improve more significantly using GL compared to typical remediation strategies used in special education classes (Saine et al., 2011). In contrast, spelling performance under solely traditional remedial teaching did not improve significantly by the end of Grade 3 (Saine et al., 2011). Main findings were that children with specific learning difficulties who were given an opportunity to replace such training with the game sessions (taking ¼ of the remediation time) reached the spelling level of their non-learning-impaired classmates.

The JLD study's findings have led to the development of this learning game. The JLD study revealed that about half of the children whose parent and his/her close relative had serious difficulties in learning to read had faced problems in early literacy. Our observations based on a very early (at the age of 3–5 days after birth) recording of event-related potentials (ERPs) revealed that when newborns were exposed to streams of sinusoidal sound pips which contained repeated standard stimuli (whose pitch was 1,000 Hz) with infrequently (12%) presented deviant sounds (of 1,100 Hz) revealing so-called mismatch negativity (MMN)-type response, we found a clear difference. Children with such familial risk who at the age of 8 years were typical learners showed reliable MMN to such deviant sounds as newborns. In contrast, the children belonging to the other half who were diagnosed with dyslexia at school age failed to show the same MMN pattern (Leppänen et al., 2010). This finding reveals that auditory insensitivity underlies a key bottleneck, making learning the earliest steps of literacy difficult (i.e., learning the sounds of the letters). These bottlenecks can be overcome with intensive use of GL (i.e., sufficient repetitive practice) as the learners are repeatedly taught to differentiate acoustically similar speech sounds (such as those represented by letters l, m, and n) as the logs reveal (Niemelä et al., 2020) and then connect these sounds reliably to their corresponding letters. This necessary first step is required to learn the basic reading/spelling skill in any transparent alphabetic orthography where the sizes of to-be-connected spoken and written items are small (i.e., single sounds/phonemes and letters/graphemes).

## Contrasts Between Transparent Writings and Non-Transparent English Orthography

**Figure 1:**
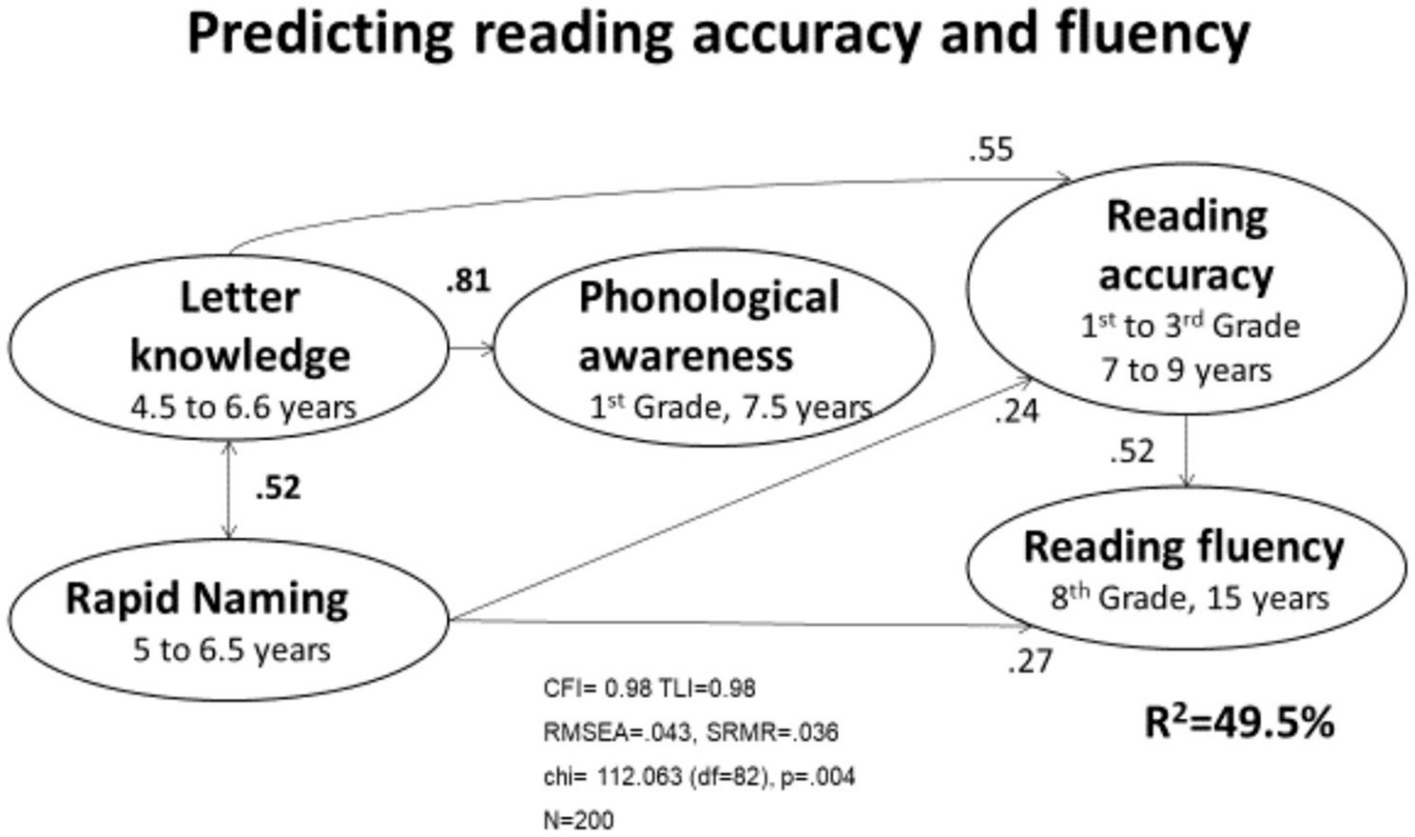
Predicting reading accuracy and fluency.

Transparent writing systems have received little attention in relation to more opaque or less consistent orthographies (especially English). While a substantial majority of reading research has focused on English, a majority of learners of alphabetic writings acquire literacy skills based on transparent orthographies. However, most reading research authors working and writing in languages with transparent orthographies (i.e., German, Italian, Spanish, and Finnish) continue to refer to research results based on reading acquisition of English. This practice continues although there is a huge difference between how English and transparent orthographies are learned. The crucial importance of phonological awareness problems, which is very central in English, is not comparably predictive in Finnish. When phonemic awareness is the most direct predictor of reading acquisition (referring here specifically to knowledge of the sounds of the letters or even well-chosen names of the letters), the predictive effect of typical phonological awareness measures disappears, as shown in Figure 1. The knowledge of letter names can predict how reading skill will be acquired with rapid naming, improving the prediction slightly.

Figure 1 reveals that reading acquisition in transparent orthographies mainly requires phonemic awareness (i.e., knowledge of the sound for each letter or grapheme of more than one letter). After the phonics knowledge of the letters of a word has been stored, the learner needs only to understand how to apply this knowledge to sound out each letter (or more than one letter grapheme) to read the sequence of letters. What is important to observe is that the early learner who has taken these two steps will be able to sound out whatever (pronounceable) sequence of letters he/she sees, independent of the length of the word or the meaning of the word. This generally results in letter-by-letter reading which may compromise the speed of the reading. If the speed of reading is then too slow, the child may have difficulty fully comprehending reading passages (even if the learner understands these words in the spoken form) due to working memory limits affecting comprehension.

Thus, the tendency of early learners of transparent orthographies to proceed *via* letter-by-letter reading/spelling leads to compromised literacy. This procedure leads to difficulties in following the meaning of the text, due to insufficient automaticity (i.e., fluency). Rapid reading makes reading enjoyable, as the child can then understand the content fully and without the need of using too much effort. Continuing to proceed using letter-by-letter reading often reduces motivation to do a sufficient amount of reading, a necessary precondition for reaching full literacy.

Learning to decode less transparently written orthographies often relies partly on meaning. This motivates the child to read more because comprehension is reached faster after the decoding skill has been acquired to the level of sufficient fluency. Understanding such orthography-related differences guides the training of literacy skills and is important for successful learning in different orthographies.

When the child is beginning to learn to read, the focus is on decoding. This early focus means that one can learn to spell and read more rapidly in transparent orthographies, because the number of the to-be-stored letter sounds is, on average, ~30 or fewer. In contrast, English has hundreds of connections between spoken and written units that need to be stored (Seymore et al., 2003). Consistent writing systems do not require the learner to know what a word means as it can be sounded out without such knowledge. The context in which the word occurs usually helps the learner to guess the meaning of a single unknown word (if the word is not in learner's spoken vocabulary).

It is important to note that in most languages including Finnish there are some aspects which are more difficult to learn than one would expect (Lyytinen et al., 2019a,b). Even typically learning children tend to make spelling errors associated with the variation of phonemic duration during the first grade. This aspect is the most difficult to learn in the spelling of Finnish. There are two phonemic lengths in Finnish: long and short, represented in their written form by repeating the letter when the sound is long. Thus, Finnish has words such as tuli (fire), tuuli (wind), and tulli (customs). The only differences appear in the context associated with repetition of the same letter. All vowels can have such a repetition. The same is possible concerning consonant letters k, l, m, n, p, r, s, and t. Related challenges usually require slightly more practice as the learner observes that one has to pay attention to not only the quality but also the duration of the sound, and for instance by sounding out the item silently to “hear” the difference to spell the word accurately for the context. Most Finnish-speaking children are sensitized to these differences (with exception of some children with dyslexia as illustrated in Richardson et al., 2003).

## Orthographic Consistency

As noted, accurate spelling of transparent orthographies is relatively easier to accomplish than that of less transparent ones. Because reading skill in non-transparent orthographies is acquired by relying on larger units stored in memory and requires acquiring orthographic word knowledge, learning becomes more demanding due to a larger number of the to-be-stored connections. This difficulty is naturally also reflected in the time needed for training the skill using GraphoLearn (GL) technology-based learning environments.

**Figure 2:**
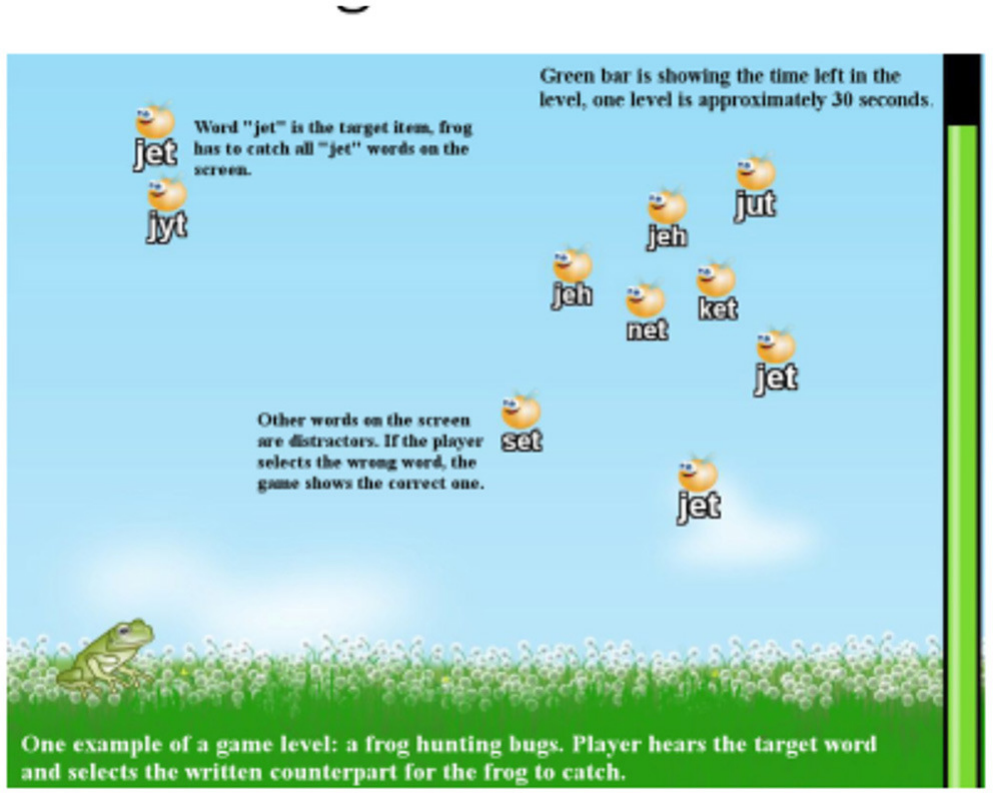
Introducing connections between spoken and written in English in the GG.

Approaching the learning of larger units such as rimes (e.g., “ong”) in non-transparently written language provides more effective training than attempting to proceed *via* smaller units because consistent connections can be learned efficiently while practically no single letter behaves consistently in English. Kyle et al. (2013) found that the gains achieved by learning small units (phoneme-grapheme connections) using GL technology were 0.47 standard score (SS) points per training hour while implementing the connection-building trials using the larger rime units (see Figure 2) leads to gains of 0.68 SS points per hour. In non-transparent writing environments, such a connection-building approach between spoken and written units was shown to be more effective than has been achieved *via* the published traditional reading interventions offered by expert remedial teachers (as we have shown also to be the case in learning transparent writing among children with dyslexia). As mentioned, the change in learning to read English per intervention hour in rime-GL game was 0.68 SS units while the comparable learning effect in a traditional intervention approach (Hatcher et al., 2006) reached gains of only 0.23 SS points per hour of training. Also, the traditional intervention was relatively time consuming and expensive. In contrast, in the GL intervention the children are training themselves, and a license to use the GG is inexpensive per learner. If licensing is made similar to that in Finland, as some kind of “state procurement,” it would be free to everyone and would include support from top experts on reading instruction such as that provided by the Niilo Mäki Institute (https://en.nmi.fi/) in Finland. This kind of expert support has been required only a few times during the more than 15 years Ekapeli (the Finnish GraphoGame) has been available in Finland.

Some alphabetic writing systems are fully consistent symmetrically to both reading and writing directions while some others behave fully consistently only for reading or spelling (e.g., not to spelling direction). German is an example for which spelling of words is not fully transparent although the reading of words is transparent. This issue does not lead to any difficulties in implementing German content in the GL-based game. In fact, the use of this GL-based game is well-documented (e.g., Brem et al., 2010, Brem et al., 2013, Mehringer et al., 2020) and is likely to be soon in wide use in Switzerland as the German GraphoGame. Neuroimaging findings also support the use of this program where good decoding gains were associated with increased activation of key left-hemisphere ventral circuits important for fluent reading (e.g., Brem et al., 2010).

The GraphoLearn technology has also been documented as efficient in helping children in France (Ruiz et al., 2017) where GraphoGame has now been in wide use already for some time. Although French has a relatively non-transparent orthography for the reading direction, French learners have been shown to acquire reading skills faster than English-speaking children learn to read in English (Seymore et al., 2003). A critical difference concerns the higher consistency of sounds represented by vowel letters in written French which lowers the burden of learning of connections between spoken and written language. This is a good example of how the content of the to-be-trained connections defines what needs to be chosen for training.

## A Detailed Description of the GL-Technology Learning Game

Figure 2 provides an illustration on how the game works when the to-be-learned connections are larger, as in the case of English. The general principle of training spelling skills using GL technology to support learning to read and spell English is that letter sequences such as ung, ang, ing, in, and ig are shown as alternatives on a display while the learner hears a sound such as /ing/ from the headphones during a trial. Such trials are repeated, helping the learners to store the most consistently behaving connections.

For a transparent orthography, the GL training usually begins with trials that contain target sounds (given *via* headphones) that are easily distinguishable phonetically (such as /a/ and /i/) so that these cannot be easily confused. At the same time, learners concurrently see on the display 2 to 3 alternative letters and must choose the one representing the given sound. The training trials progress to presenting sounds representing phonemes acoustically closest to each other such as those represented by letters l, m, and n. After minimal pairs of the items most difficult to differentiate are successfully learned, the next trials present a larger number of alternatives from which to choose, to assess the reliability of the learning. The next step is word assembly; the learner is shown written items containing sequences of several letters (forming a syllable or even a short word). With this approach, the learner begins to understand how the sounds of the letters correspond to the written stimuli when more than one letter is sounded out and assembled into a word represented by these letters. When such syllable or word-size item is presented orally as a target spoken unit, the learner succeeds by choosing the correct alternative; thus, s/he demonstrates an understanding of the alphabetic principle. This can (and has) been done for a variety of languages. In general, the processes included in the GL method demonstrate that the training supports accurate and fluent spelling not only of the smallest units (phoneme/grapheme) but also of syllables or words after the child has learned sounds associated with single letters as well as larger units.

In a sense, the game introduces a type of simple dictation task. It is made easier by requiring that the learner choose from the appropriate alternative written items the one that is spelled accurately according to the sound received from the headphones in each trial. This very simple principle is working for all levels; students learn of the connections, assembling the smallest items to form syllables and words and then developing fluency (speed) at each level. The final level—supporting reading comprehension—is under development.

In order to demonstrate the use of the GL method in transparent orthographies beyond Finnish, the following sections will discuss its application to Chinese, Japanese, and sub-Saharan African languages. In these very dissimilar languages, the GL method has been shown to be helpful for children with and without learning problems.

## Supporting Early Steps of Literacy Learning of Asian Orthographies

Chinese writing presents a different situation compared to European languages as the orthography is non-alphabetic. Chinese and Japanese have a consistent first step in written language acquisition by using a sound–symbol system to get children started. In Mainland China, such a first step is similar to alphabetic writing systems. Children learn to connect sounds and written alphabetic symbols; later, they will learn the characters used in writing. This system, Pinyin, is used to introduce children to principles of reading by teaching the Chinese sounds (phonemes) with corresponding Chinese symbols. Pinyin GraphoGame is in wide use in Mainland China after having been documented as effective (e.g., Li et al., 2016). Pinyin sounds to symbols can be easily acquired by using the GraphoGame (see (Graphogame.com).

In Japanese, the Kanji writing system is close to Chinese characters, while Hiragana/Katakana writing behaves similar to Pinyin but uses larger units. This more alphabetic orthography, which connects spoken to written units, is used in Japanese to spell foreign names. The alternative way of using analogy for Kanji-related sounds or sounds of Chinese characters does not work as accurately in helping to sound out foreign names. Individual spellers frequently wind up using different ways to write the same names when Chinese characters or Kanjis are used for that purpose.

As the Pinyin system provides a close association with the way alphabetic writings work, so also do Japanese Hiragana and Katakana, which are almost the same as syllable-like units of transparently written alphabetic orthographies (i.e., instead of being represented by a sequence of several letters, here only one written symbol represents a syllable size sound unit).

Although Hiragana spellings may be the easiest to learn, the system is more limited than alphabetic orthographies. The use of the GL technology has been documented in an article published in Japanese (under the supervision of Uno Akira) but not translated into English at this time. Spelling in this system is as easy to learn as the symmetrically consistently written orthography of Finnish, which is illustrated in detail below. Skilled readers of Japanese must use both Hira/Katakana and Kanji writings in day-to-day reading in Japan. Chinese readers are using only Chinese characters (which are like Kanjis) without relying on the Pinyin system, which is used only as an introduction to reading skill. A different approach to reading is present in Hong Kong where Pinyin learning is not included in reading instruction. Children are generally exposed to alphabetic writing of English that connects written symbols to sounds, a comparable connection between written symbols and sounds which Pinyin introduces to Mainland children who are not exposed comparably to English.

As stated above, Chinese and Kanji symbols represent more than sounds. These languages may contain cues for sounding out words, bringing the reader often close to the meaning of the sentence because some of the symbols are similar to pictures (called pictographs) of the concepts they represent. Research is continuing using the GraphoLearn technology for teaching Chinese writing beyond the use of Pinyin, and it is currently being refined.

## GL for Chinese Spelling

Compared to learning to read and write alphabetic languages, learning to read Chinese is much more of a challenge. The Chinese orthography is often described as logographic or morphosyllabic. The basic graphic unit in Chinese is a character. Each Chinese character, morpheme, and syllable share a one-on-one correspondence. There are about 3,500 simplified Chinese characters in daily use in Mainland China and about 4,500 frequently used traditional characters in Taiwan. While Chinese characters are well-known for their visual complexity, spoken Chinese has a relatively simple phonological structure. A syllable consists of an optional initial and final sound segment (e.g., onset and rime). Mandarin contains only 403 syllables, and the total number rises to about 1,200 distinct “tone syllables” when the four tones, or voice inflections—high, rising, low then rising, and falling—are taken into account.

The script–meaning relationship in Chinese is similar whereas the script–sound correspondence is arbitrary. One advantage of the logographic and morphosyllabic nature of Chinese is that the same script can be used with a large population in which people speak different dialects. The disadvantage is also obvious. Learning to read Chinese depends on memory and integration information about orthography, phonology, and semantic meaning of thousands of characters in the early stages of reading Chinese. Thus, schoolchildren in Chinese need a longer period to complete reading acquisition.

Considering the less reliable orthography–phonology connection in the Chinese writing system, Pinyin (a consistent and transparent phonological coding system) is used to provide the sound information of characters for beginning readers. The Pinyin system roughly corresponds in appearance to the Western alphabet. It represents single phonemes as in alphabetic scripts, but it is taught in a syllabic way, dividing a syllable into onset and rime. First graders in mainland China usually learn Pinyin in the first 8 weeks of school. Following initial instruction, Pinyin is written above new characters during reading for young children as they move on to character reading.

An increasing number of studies have shown that Pinyin instruction enhances both phonological awareness (e.g., Chen and Yuen, 1991; Cheung et al., 2001; Xu and Ren, 2004; Ren et al., 2006; Shu et al., 2008) and character reading (e.g., Shu and Liu, 1994; Siok and Fletcher, 2001; Lin et al., 2010) in Chinese children. Mastering Pinyin is a crucial component of Chinese literacy development in early school years (e.g., McBride-Chang et al., 2012). Children typically receive intensive Pinyin training for 10 weeks at the beginning of grade one (Wu et al., 1999). This typical 10-week Pinyin instruction may not be sufficient for children with poor reading skills, given that some children still experience difficulties in acquiring Pinyin skills at the end of the first grade (Li et al., 2016). In order to help these Chinese children, especially poor readers, to improve Pinyin skills, we have developed a Pinyin GraphoGame and examined the effects of it with typical intervention designs.

Li et al. (2016) provided an intervention for first graders who performed poorly with traditional teaching of Pinyin instruction as provided by their schools. These children used Pinyin GL-based game for a 4-week period. All played the game on computers at home. Compared to their peers in a control condition, the children in the training condition outperformed controls on both Pinyin reading accuracy and fluency. In another study, Li H. et al. (2017a) provided an 8-week period of having Pinyin GL available for first-grade children with poor Pinyin skills. This intervention improved Pinyin reading accuracy for all participants. Yue et al. (2019) made the Pinyin GLe intervention available for 8-week- to 6–12-year-old children diagnosed to represent both reading disabilities and ADHD. Results showed that the intervention improved the children's phonemic awareness and Pinyin recognition. These results indicate that the Pinyin GraphoGame is a cost-effective method to enhance Pinyin and literacy outcomes for underprivileged children in China.

Although the research discussed has provided evidence that Pinyin is beneficial to Chinese readers as a starting point, Pinyin is not a common writing system for formal documents. Chinese children must learn characters, and to do so they must store thousands of connections between the writing symbol and sound to accomplish basic reading skills. Successful Chinese character acquisition depends upon being able to activate and maintain three representations, visual (i.e., character), verbal (i.e., pronunciation of the character), and semantic (i.e., meaning of the character), and to form a new association between the first two in long-term memory. Indeed, it has been suggested that paired associate learning may be particularly important for children learning to read Chinese (Ho et al., 2006), precisely because of the relatively arbitrary nature of verbal–visual correspondences in the pronunciation of some Chinese characters, especially for learning to read irregular characters for which no clue to pronunciation is available.

The GraphoGame may be an effective intervention tool to effectively support Chinese children to learn the necessary basics for acquiring reading and spelling skills in Chinese. The development of this program will take time because the number of connections one has to store is many times larger than learners need for acquiring the skills needed in transparent orthographies. It is also substantially larger than required for learning the connections between spoken and written English.

## GraphoLearn Research of Reading and Spelling Skills in Africa

In Africa, teaching reading and writing is interesting and complicated. Most African countries begin reading instruction using a carefully chosen language from the typically huge number of local languages in each country. The local Sub-Saharan languages have transparent writing, which frequently is present solely in written form in the Bible translated into the native language. Often, this language can be one language (such as Kiswahili in Tanzania) or several (such as the seven languages in Zambia that have been chosen to be used during first grades for teaching). These languages become the foci of reading instruction for these children with needs to be used for the initial literacy learning in home languages (Lyytinen et al., 2015a, 2019a,b).

Prior to 2000, teachers in today's Zambia were instructed to learn to read English at the beginning of their own school career. The result of this training is that many of these teachers, if not most of them, have not learned how to teach fully transparent African local languages. In English, the sound of a vowel can depend on the context for the word while in fully transparent in Sub-Saharan African orthographies each letter consistently represents only one phoneme in any context. Thus, in African orthographies reading instruction is built *via* the connection-building approach where the learner has sufficient exposure to the sounds of the language s/he is learning to read and the corresponding written units (Lyytinen et al., 2019a,b). The traditional reading instruction used in Sub-Saharan African countries is based on teaching English has not been successful teaching reading in a transparent orthography (Lyytinen et al., 2019a,b). Using this approach in Zambia has resulted in almost every learner requiring additional help to learn to read and write mainly because of the mismatch between teaching a non-transparent orthography (English) to teach a totally different type of orthographies (Sampa et al., 2018). As a result, very few children have learned the basic foundations of reading prior to third grade (i.e., the time when the reading instruction focusing on local languages ends), resulting in poor performance on the main measure of academic achievement; the Early Grade Reading Assessment (EGRA) (Sampa et al., 2018).

A very serious problem in many places of Africa is that the children in most need of help live outside large cities, making access difficult. The finding that practically all families own a sufficiently usable mobile phone in which the game works allows the game to be distributed. One challenge still remains, however. We need to find a way which would work for providing sufficient instruction to families and teachers on how to use the digital learning environment, especially in cases where families live outsides cities and do not have proper access to the Internet. For this reason, we are collaborating with internet network-building experts to provide guidance from a distance by well-trained experts in the country, such as those who conducted the validation research in each country.

For the purpose of providing experts in GL in Sub-Saharan Africa, together with the local experts we helped to create the Center for the Promotion of Literacy in Sub-Sahara (CAPOLSA) in the context of University of Zambia (UNZA) in Lusaka. At this center, there are several PhDs who have published the set of 3–4 international papers each according to Finnish standards for their PhD theses which they have defended in Finland. All of these publications are based on experimental studies made for validating of the efficiency of the game to support learners in learning to read. The experts in CAPOLSA are ready to help not only in Zambia but also in other Sub-Saharan countries.

Research conducted for one of the dissertations has found that illiterate parents are readily able to learn the basic reading skills in a manner similar to that through which their children learned using the GraphoLearn technology (Nshimbi et al., 2020). Thus, it may be that the impact of such a simple learning game, which works on inexpensive phones owned by most families in Africa, will involve entire families in the near future.

The GL system, tailored to the specific language, provides basic phonics instruction in a very concrete, consistent, and effective way using widely available, inexpensive mobile phones [e.g., Jere-Folotiya et al., 2014; see for a review of our related African studies, Ojanen et al. (2015a,b) and Lyytinen et al. (2019a,b)]. This program is most advantageous when children use the GL game at the same time as their teachers are teaching effective phonics instruction in their native language using the program (Jere-Folotiya et al., 2014). When the name of each letter is related to the sound of that letter (if chosen carefully as done in Finland), children readily learn to read when exposed to letters and sounds within the preschool class environment even when not taught formally how to read. When a child fails to store letter names under such conditions in preschool, s/he often face problems in learning to read (e.g., Lyytinen et al., 2015a). Ojanen et al. (2015a,b) and Lyytinen et al. (2019a,b) provide summaries of the results of African studies associated with the use of the GL technology. Following the validation of the program, the technology is now made available asGraphoGame (GG, see graphogame.com). It is hoped that this program will be implemented in more schools compared to the typical instruction used unsuccessfully in Zambia.

In a writing system such as that of Finnish or sub-Saharan African (e.g., Bantu) languages, the connections between spoken and written units behave consistently at the phoneme–grapheme (letter) level. The Finnish Ekapeli Alku (initial steps of the Finnish GraphoLearn) can be used to teach African local languages such as those belonging to the Bantu group because the sounds of the letters and syllables work the same way. Learning is made simpler if the instruction is organized by first learning letter names (but not those used in English, which is a misleading approach used in some countries of Africa). The names of letters in Finnish writing have been chosen to represent relatively close matches to the sounds of those letters. Typically, the letter symbols are present on the classroom walls of the preschool environments. Thus, these visual cues these letters represent encourage the child to wonder what these figures are called, which motivates them to learn the names of all such figures. These letter-figures often are connected to pictures of familial objects such as animals whose names children know. These figures are typically located next to the letters on the classroom walls in such a way that the child sees a picture representing a picture whose name starts with the letter adjacent to it. This practice ensures that children learn most of the names of the Finnish letters before entering school. Those few who have not learned them 1–2 years before they enter school at age 7 face more difficulty when learning basic reading skills (Lyytinen et al., 2015a). The JLD study has revealed that the age at which a child has spontaneously learned the letter names predicts accurately the time s/he needs for learning to read (Lyytinen et al., 2015a).

Because of the difficulties these Zambian children are experiencing, the GL-based research in Zambia has become a priority and a large number of studies have supported the program for instruction [see a review of the results, see Ojanen et al. (2015a,b) and Lyytinen et al. (2019a,b)]. Because of the difficulty these children are experiencing, we are moving as fast as possible from research to the distribution of the GraphoGame learning environment for which we have given rights to the Grapho Group Ltd company. The company now uses the name GraphoGame for the learning environment because the owners of the intellectual property rights (University of Jyväskylä and Niilo Mäki Foundation) transferred the rights to the company to provide international delivery of the digital learning environment. The strategy of the Grapho Group company is to distribute the game without seeking for profits to countries UNESCO defines as poor. Moreover, the company has agreed to make it available as widely as possible to all in need once the research has documented the efficacy *via* its GraphoLearn research process for any particular country/language. Therefore, the program (GraphoGame) will soon be available (e.g., in Tanzania and Namibia, after we have documented how GL can also most efficiently be delivered in rural Africa).

The next goal in Africa is to start using GraphoLearn technology for teaching second-language reading of English, French, Spanish, and Portuguese according to the prevailing preferences of each country. Naturally, the final goal of all reading is full literacy, comprehending different types of texts which one reads. This final version of the game is under development, first using the most widely spoken alphabetic languages.

## Use in Latin America

Several ongoing projects at the Haskins Global Literacy Hub (HGLH) at Haskins Laboratories, a Yale University and University of Connecticut-affiliated research institute, have been exploring the efficacy of GraphoLearn in English speaking learners. In one NIH-funded treatment study, GL is paired with an in-school treatment program (EMPOWER developed by Lovett and colleagues) for remediating reading difficulties in children and uses neuroimaging at frequent intervals to better understand how treatment moderates brain circuits for literacy and why some children respond better than others despite similar behavioral profiles. A second study, also funded by the NIH, involves a larger number of children participating in the Healthy Brain Network study at the Child Mind Institute in New York, each with intensive gene-brain-cognitive profiling. The impact of GL on different subtypes of struggling readers with varied comorbidities is being investigated. Moreover, GL is serving an important function in three HGLH projects associated with the COVID-19 crisis. In one NSF-funded project (Pugh and Hoeft directors), GL is being used for groups of children at risk for the expected COVID reading slide (given up to 6 months during which students are unable to attend in-person schooling). Online reading assessments before, during, and after 12 weeks playing GL are assessing the mitigating effects of ED tech on reading. Finally working with GraphoGame, the HBLH has been able to provide free access to the game in the U.S. (English), Brazil (Portuguese), and Argentina (Spanish) during the COVID crisis. Thus, the use of GL to serve children at need is a major priority for the hub.

## Learning to Spell Non-Transparent English as a Second Language

In many, if not most, countries English is the second language a child learns. Typically, reading in English is taught through the use of whole words, a practice that has been successful in Finland. A learner who knows the sound and meaning of a spoken word in a foreign language can store the written form of that word. As an alternative way to learn to read a foreign language, our game program introduces reading in the foreign language using optimal phonics. In the game, whole words can be used together with smaller units such as rimes, following the more traditional method for learning to read a foreign language because these learners already have a level of phonological awareness, an understanding of the alphabetic principle, and a basic understanding of language (that it is made up of sounds, words, and phrases and sentences) from their native language.

Whole-word learning can be encouraged especially if the L2 reading starts later than first grade. At that point, the child is readily able to store spoken words, the meaning of which s/he has learned and can store the written word in one's lexicon. When the written word is presented together with the spoken word, young learners acquire the accurate sounding of the foreign word more easily than older learners, likely because they store the sound of the word simultaneously with the meaning. Thus, beginning to learn a foreign language begins early in life and learning to read using a phonetic approach. For this purpose, GraphoLearn technology is ready to be tried. Versions of English (e.g., Kyle et al., 2013), French (Ruiz et al., 2017), German (e.g., Brem et al., 2010), Spanish (Rosas et al., 2017), and Portuguese (e.g., Carvalhais et al., 2020) are available for GL studies for the support of learning to read a second language.

## Concluding Remarks

We have developed GL-based learning tools supporting acquisition of the basic reading skills which can run on inexpensive smartphones and tablets (Android and iPhone/iPad) and computers using Windows and Mac operating systems. These tools have been shown to work in tens of countries to help children to learn to the basic reading and spelling skills. Beginning in 2020, more than 10 countries widely use the program under the label GraphoGame.

The new challenges associated with the validation research include countries like India that has a large number of different writing systems or China whose writing systems are exceptionally complex. Importantly, children in China and India also can use GL to help practice English as a second language, so that they can acquire basic reading skills of English much faster than with more traditional school instruction. This improvement, however, requires that they use GL/GG optimally. Excellent suggestions on implementation and usage of GG are available from the meta-analysis published by our Norwegian colleagues (McTigue et al., 2019).

When attempting to use GL/GG to help children with dyslexia, it is important to note that the training must be started prior to school entrance. Thus, whatever risk factors (such as familial risk, delayed development of spoken language, poor instruction, difficulties in storing the letter names) must be identified before entering school to be able to start preventive training at the optimal time. Using the GL learning environment as a dynamic assessment method during the 1st days of school if the child is at risk of familial dyslexia has been shown to be very successful for noting whether one needs it and at the same time for helping the child to learn (unpublished Zambian PhD thesis by Munachaka). The use of this dynamic assessment (DA) can be helpful for differentiating between educational insufficiencies or biological etiologies for reading difficulties. When there are poor instructional conditions, the use of DA soon reveals that children would be able to learn efficiently if instructed optimally, but if the learner is facing biological difficulties even a short use of the game as DA tool shows that the readiness of learning is compromised as shown by the mentioned data from Zambia. Children he followed for 6 years from school entry showed improvement despite poor teaching. However, when the GL-based dynamic assessment was used at school entry, it was shown that children with poor readiness to store the sounds of letters when using the game in DA mode did not progress in learning to read during the following 6 years of school instruction (i.e., could be defined learners with dyslexia).

If learning does not proceed relatively rapidly under GL/GG-based training during the 1st days of school, our recommendation is that the use of the learning game be continued for about 10–15 min sessions at least 2–3 times per day on consecutive days weekly. Its use is recommended to be continued at least until progress is made in the acquisition of the connections between spoken and written units so that the child feels her/his performance equals the progress of typical learners among the first grade classmates. This type of start may allow at-risk children to avoid facing negative learning experiences when they become aware that they are reading more poorly than their peers. Negative early experiences for reading acquisition affect not only training for literacy skills and interest in reading but also all learning in school. Our program is designed to avoid these secondary difficulties which can often lead also to emotional problems.

## Author Contributions

HJ wrote most of the text. MS-C cleaned it and made it written with perfect English. HL wrote the part which is associated with learning to read Chinese. KP helped adding content concerning the part of activity led by his network and accepted the content as it is. UR cleaned what she saw being in need of doing that. All authors contributed and approved the submitted version.

## Conflict of Interest

The authors declare that the research was conducted in the absence of any commercial or financial relationships that could be construed as a potential conflict of interest.
